# Raman microscopy of porcine inner retinal layers from the area centralis

**Published:** 2007-07-12

**Authors:** J. Renwick Beattie, Simon Brockbank, John J. McGarvey, William J. Curry

**Affiliations:** 1 Respiratory Medicine Research Cluster, Institute of Clinical Science, Queen's University Belfast, Belfast, Northern Ireland; 2 Centre for Clinical Raman Microscopy, Institute of Clinical Science, Queen's University Belfast, Belfast, Northern Ireland; 3 Ophthalmic Research Centre, Institute of Clinical Science, Queen's University Belfast, Belfast, Northern Ireland; 4 School of Chemistry and Chemical Engineering, Stranmillis Road, Faculties of Medicine and Science and Agriculture, Queen's University Belfast, Belfast, Northern Ireland

## Abstract

**Purpose:**

To characterize the Raman spectra of porcine inner retinal layers, specifically, the inner nuclear, inner plexiform, ganglion cell, and nerve fiber layers.

**Methods:**

Raman microscopy was employed at three excitation wavelengths, 785, 633, and 514 nm to measure Raman spectra in a high resolution grid across the inner layers of 4% paraformaldehyde cryoprotected porcine retina. Multivariate statistics were used to summarize the principal spectral signals within those layers and to map the distribution of each of those signals.

**Results:**

The detected Raman scattering was dominated by a signal characteristic of the protein population present in each layer. As expected, a significant nucleotide contribution was observed in the inner nuclear layer, while the inner plexiform layer displayed a minor contribution from fatty acid based lipid, which would be characteristic of the axonal and synaptic connection resident in this layer. Blood vessels were readily characterized by their distinct heme-derived spectral signature, which increased at 633 and 514 nm excitation compared to 785 nm. Discrete isolated nucleotide signals were identified in the ganglion cell layer, while the nerve fiber layer exhibited a homogenous profile, which is indicative of its broadly uniform axonal and cytoplasmic Muller cell components.

**Conclusions:**

The present study demonstrated the potential of Raman microscopy as a tool to study the biochemical composition of pathologically normal retina. Specifically, the method allowed a unique method of analyzing the network of neurons involved in relaying information from the photoreceptor population to the ganglion cell derived nerve fiber layer. The study has demonstrated the ability of Raman microscopy to generate simultaneously information on a range of specific biochemical entities within the stratified normal retina.

## Introduction

Raman microscopy produces detailed spectral information at high spatial resolution (1-3 μm) about the chemical and physical characteristics of a wide range of biological materials. The technique requires minimal sample preparation thus minimizing potential artifact generation which may be introduced by extensive preparative techniques; additionally sample analysis is not destructive. It has been used to study the chemical and physical composition of a range of biochemicals characteristic of the retina, including proteins [1], fatty acid based lipids [2], DNA [3], saccharides [4], cytochromes/hemes [5]. The final two biomolecules are often present as minor components (in terms of percent by weight); however, when an excitation wavelength matching a UV/Visible absorption band is used some Raman bands from the absorbing molecule are greatly enhanced, increasing the sensitivity of Raman spectroscopy. The measurement of these enhanced Raman bands is known as Resonance Raman Spectroscopy (RRS). This approach enables the detection of certain minor components even in the presence of a dominant matrix. Combining Raman scattering with microscopy allows the investigation of the variation on a micrometer scale of the vibrational spectrum (and thereby the study of the chemical and physical variation) within a sample. Consequently, Raman microscopy has been used to study a wide range of biomedical samples and to assess the biomolecular characteristics of normal and pathology specimens [6]. Pathological analysis of the eye, in particular the cornea and interior ocular fluids and tissues, are viable targets for in-situ Raman spectroscopy analysis [7], while RRS has been extensively used to study carotenoid content in human retina [8].

Since the retina is arranged in architecturally distinct layers, each comprised of specific cytoplasmic and nuclear compartments of distinct cell types, it is particularly amenable to Raman analysis. For example, the outer plexiform layer (OPL) contains the synaptic zone between the photoreceptor cells and the second order postsynaptic neuronal horizontal and bipolar cells. The inner plexiform layer is also a synaptic zone, where the neuronal cells spanning from the OPL synapse with third order amacrine and ganglion cells. The inner nuclear layer (INL) is comprised of the cell perikaryia, while the ganglion cell layer (GCL) contains perikaryia and gives rise to the nerve fiber layer (NFL), consisting of neuronal processes that exit the eye via the optic disc to form the optic nerve. The nuclei of the radial Muller cell are located in the INL, they form extensive cytoplasmic processes that pass through the outer nuclear layer (ONL) and ganglion cell and nerve fiber layers, generating the outer and inner limiting membranes; they are the principal glial retinal cell type. The complex communication pathways between Muller cells, retinal neurons, and the vascular system indicate that they play a pivotal role in retinal physiology [9].

The present study is part of a series investigating the use of Raman microscopy to characterize the biochemical and biophysical composition of porcine retina. In a previous paper we reported the use of Raman microscopy to map the photoreceptor layer, specifically, the outer nuclear layer (ONL) and photoreceptor inner and outer segments (PIS and POS) [10]. Proteins, including cytochrome C, and DNA were observed and their distribution profiled on a Raman map. The present paper completes the characterization of the porcine area centralis, concentrating on inner retinal layers, which are specialized for the transfer of electrical impulses from the photoreceptor layers to the nerve fiber layer.

## Methods

### Tissue specimens

Porcine (White landrace) eyes from 2 pigs were enucleated at the abattoir (Stevenson and Co, Cullybackey, Co Antrim, Northern Ireland) within 30 min of death and transported to the laboratory in ice and immersion fixed in buffered 4% (w/v) paraformaldehyde (24 h, 4 °C), prior to initial cryoprotection in 5% (w/v) sucrose/phosphate buffered saline (PBS, 0.05 M Na phosphate, 0.14 M NaCl, pH 7.4) with storage in 30% (w/v) sucrose/PBS containing 0.01% (w/v) sodium azide. Cryostat-derived retinal cross-sections (20 μm) were prepared from the area centralis [11] and placed on quartz slides. The Raman measurements were repeated on two independent samples.

### Control reagents

A number of control reagents were selected to assist with the identification of the detected retinal Raman profile dataset. The Raman signal was recorded from TLC grade (>99%) melatonin (catalog number 63610, Fluka, Buchs, Switzerland) in a saturated ethanolic solution. The Raman spectrum of a saturated aqueous solution of TLC grade (98%) L-tryptophan (catalog number T0254; Sigma Aldrich, St Louis, MO) was recorded.

### Raman microscopy

The Raman spectra were recorded using a Jobin-Yvon LabRam HR800 Raman microspectrometer. The spectra were generated from sections of cone rich retina (area centralis) using 514 (10 mW), 633 (20 mW), or 785 nm (100 mW) lasers as excitation sources, focused on the sample with a 50x objective in a nonconfocal arrangement (confocal hole diameter 800 μm), with the laser focused to about a 3 μm diameter spot. The microspectrometer was fitted with a 1 μm step xyz stage. A 300 groove mm-1 diffraction grating was used with 785 and 633 nm excitation, while a 600 groove mm^-1^ diffraction grating was used in conjunction with 514 nm excitation. Spectral resolutions were 10, 12, and 8 cm^-1^ with 514, 633, and 785 nm excitation, respectively. All optical images and spectral maps were recorded and processed using the Labspec software (Jobin-Yvon, Villeneuve d'Ascq, France). Statistical analysis was performed using Simca P8.0 (Umetrics, Umea, Sweden). Raman spectra were acquired at 3 μm spacing intervals for a depth of 80 μm across the retinal layers and for a length of 30 μm parallel to the layers. The maps from each wavelength were recorded sequentially at the same location, with the brightfield image checked between each map, and after the final map, to confirm that the stage had returned to precisely the same position (within 1 μm of the original position). Following Raman data acquisition the tissue section was stained using a standard hemotoxylin and eosin protocol to enable histological assignment of the retinal layers.

The spectrum of the quartz slide was subtracted from each spectrum simultaneously using the LabSpec software. In the spectral subtractions, any residual intensity arising from the sucrose medium was subtracted by normalizing about the doublet of bands at 1069 and 1135 cm^-1^, and about the band at 845 cm^-1^.

### Multivariate analysis

As previously described, the non-Raman background was removed and the spectra normalized about the total area of the spectrum prior to analysis by multivariate statistics. The spectra were assigned to the different retinal layers following analysis of the analogous histologically stained sections that had been employed to collect the Raman spectra, and mean-centered. Principal Component Analysis (PCA) was completed for each layer using Simca P8.0 (Umetrics, Umeâ, Sweden). The scores for the first two components of each layer were used to determine the main component spectra. High and low score spectra were averaged and used as model spectra to construct a Raman map of component distribution by linear combination of elements (LCE) in the Labspec software. LCE simply involves summing the various model spectra using linear functions until the result matches the sample spectrum.

## Results

As previously reported for the outer retina [10] the majority of the spectra displayed no evidence of resonance enhancement at any of the three wavelengths employed, therefore the spectra acquired at 633 nm were considered typical, except for those instances where some enhancement was evident at other wavelengths.

### Plexiform layers

**Figure 1:**
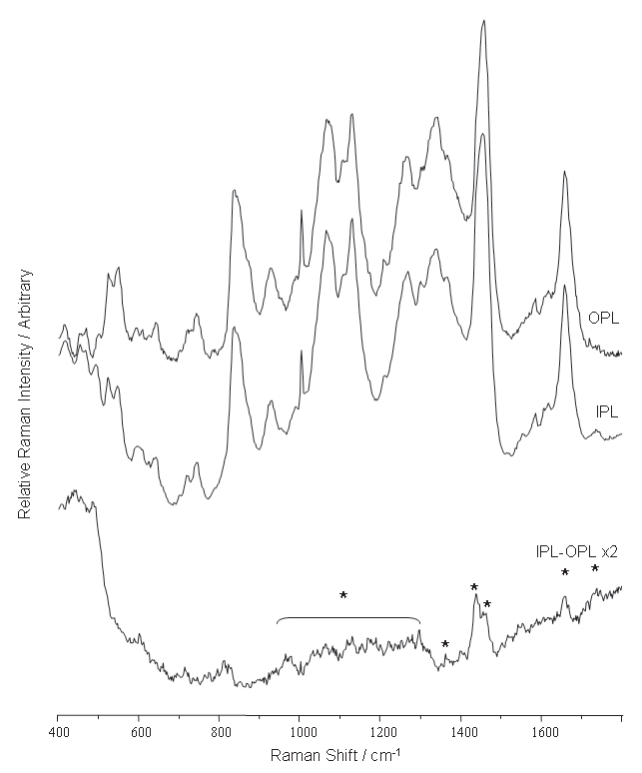
Raman spectra of the outer and inner plexiform layers. The spectra, which were acquired using 633 nm excitation, reveal the biochemical similarity between the spectra of the inner (IPL) and outer (OPL) plexiform layers. The only significant spectral difference between the two layers appeared to match the profile of fatty acid based lipid (asterisk).

The average spectra of the OPL and IPL (Figure 1) were dominated by protein and as expected there was no evidence of bands characteristic of either DNA or cytochrome C; these spectra were previously observed in the ONL and PIS [10]. The subtraction spectrum displayed positive bands at 1730, 1660 and 1440, and a broad region from 900-1330 cm^-1^, which is typically indicative of fatty acid based lipids (Figure 1).

### Inner nuclear layer

**Figure 2:**
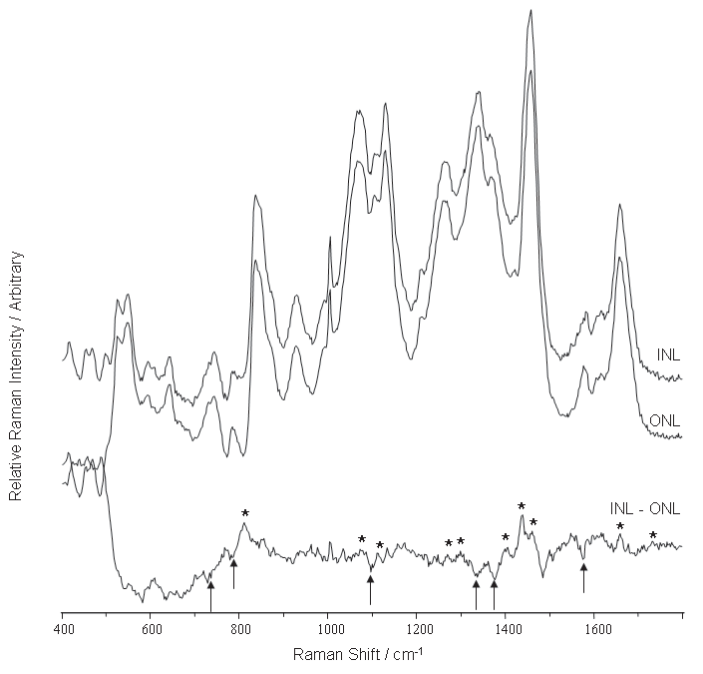
Raman spectra of the inner nuclear and outer nuclear layers. The spectra, which were acquired using 633 nm excitation, reveal the difference between the spectra of the inner (INL) and outer (ONL) nuclear layers. The positive bands in the subtraction spectrum are related to fatty acids (indicated by asterisk) and negative bands related to DNA (indicated by arrows).

The Raman spectrum of the INL displayed distinctive bands arising from DNA modes, with intensities at 1580, 1375, 1340, 1090, 785, and 730 cm^-1^, a pattern characteristic of this nuclear layer (Figure 2). However, there was a significant decrease in the relative contribution of a DNA signal to the overall intensity of the spectrum relative to that observed in the ONL; this was evident in the subtraction spectrum (Figure 2). This observation correlates with the corresponding decrease in the number of nuclei resident in the INL. The series of positive bands in the subtraction spectrum at 1735, 1660, 1460, 1440, 1300, 1270, 1130, 1065, and 800-900 cm^-1^ are suggestive of an increased fatty acid based lipid component in the INL compared with the ONL.

### Ganglion cell layer

**Figure 3:**
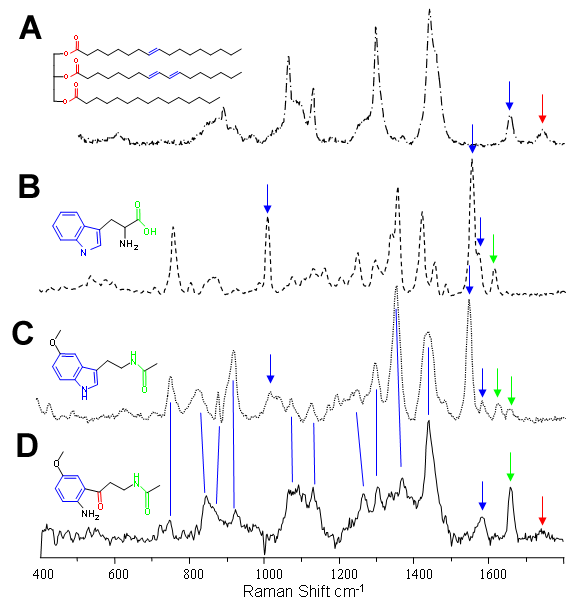
Inner plexiform layer signal compared with reference compounds. All spectra were recorded using 633 nm excitation. Raman spectra of (**A**) a typical triglyceride (porcine adipose), (**B**) tryptophan, and (**C**) melatonin are compared with (**D**) spectral signal found in ganglion cell layer. The spectrum in **D** is a scaled spectral subtraction of the ganglion cell layer signal from that of the inner plexiform layer. The subtraction was scaled to preclude negative bands; all common spectral features were removed. Additionally, color highlights, marking the chemical structures of the compounds with specific chemical bonds, correspond to colored arrows, indicating the region where spectral features appear. These, in turn, correspond to vibrational normal modes involving these bonds; blue (aromatic/olefinic), green (amide) and red (carbonyl). The scaled subtraction spectrum exhibits many bands in common with those in the reference spectra, but significant differences may tentatively be assigned to a combination of fatty acid and the oxidation product of melatonin, N^1^-acetyl-N^5^-methoxykynurenamine (structure shown).

Comparison of the average spectrum of the GCL (excluding the blood vessels) with the IPL revealed some complex changes between these two layers, which made interpretation tricky. Therefore, a scaled subtraction was performed, where the subtraction was stopped just before any negative bands appeared in the subtraction spectrum (Figure 3D). Positive bands in the subtraction spectrum IPL-GCL occurred at 1740, 1660, 1580, 1460, 1265, 1085, 1065, and 935 cm^-1^. The presence of the band at 1580 cm^-1^ does not correlate with fatty acids, and the relative intensities of the bands was not typical of fatty acids, an example of which is shown in Figure 3A. The occurrence of a band in the 1500-1600 cm^-1^ region is often associated with aromatic molecules. Many of the bands overlap those found in the Raman spectra of tryptophan and melatonin (Figure 3B,C), with additional bands appearing at frequencies typical of carbonyl/amide groups. A possible candidate assignment for this spectrum is N^1^-acetyl-N^5^-methoxykynurenamine, the metabolic breakdown product of melatonin.

### Blood vessels

**Figure 4:**
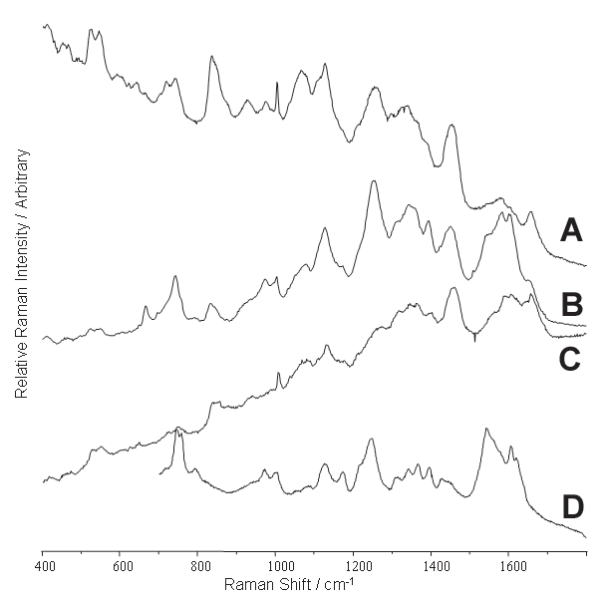
Raman spectra of the blood vessels. The spectra were acquired using (**A**) 785 nm, (**B**) 633 nm. and (**C**) 514 nm excitation. **D**: Raman spectrum of clotted porcine blood. The positions of the various bands within the retina display features at positions matching those of the clotted blood, although the relative intensities are probably altered, due to a difference in oxidation state.

The spectrum obtained from blood vessels at the periphery of the GCL/NFL interface was recorded at three excitation wavelengths (Figure 4A-C) and exhibited characteristic heme bands (c.f. spectrum of venous blood at 633 nm, Figure 4D), with a characteristic pattern of two groups of three bands at 1500-1650 and at 1320-1420 cm^-1^, the exact position and relative intensity of which are sensitive to the oxidation and conformational states of the heme [5,10]. The intensity of the heme bands relative to the protein bands was extremely wavelength dependent, with the weakest heme signal being observed at 785 nm, the strongest at 633 nm, and the intermediate intensity at 514 nm (Figure 4).

### Nerve fiber layer

**Figure 5:**
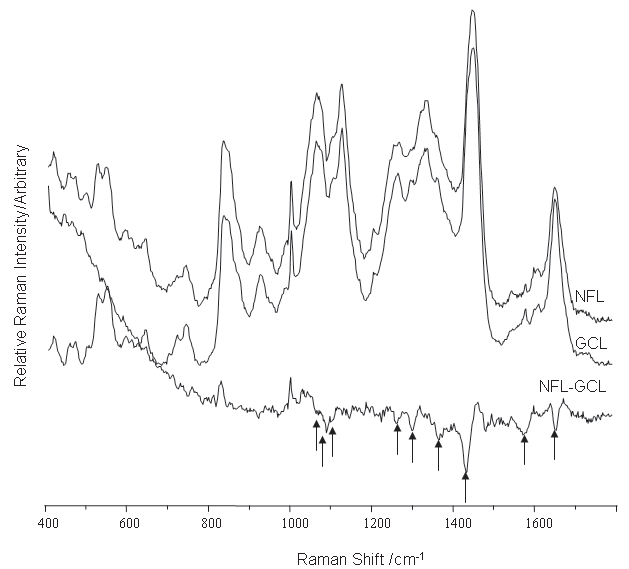
Raman spectra of the nerve fiber layer and ganglion cell layer. The spectra were acquired using 633 nm excitation. Arrows highlight the major bands that decrease on moving from the ganglion cell layer (GCL) to the nerve fiber layer (NFL) are highlighted (arrows). These bands match the differences found between the inner plexiform layer and GCL (Figure 4).

Comparison of the Raman spectra for the NFL with the GCL (Figure 5) revealed an extremely complex series of changes between these two layers. This may reflect the differing protein and lipid population present in the two layers. The NFL, which is comprised of unmylenated neurons is less complex and has a more uniform layer than the GCL, which is comprised of a wide range of cell types.

### Principal component analysis

**Figure 6:**
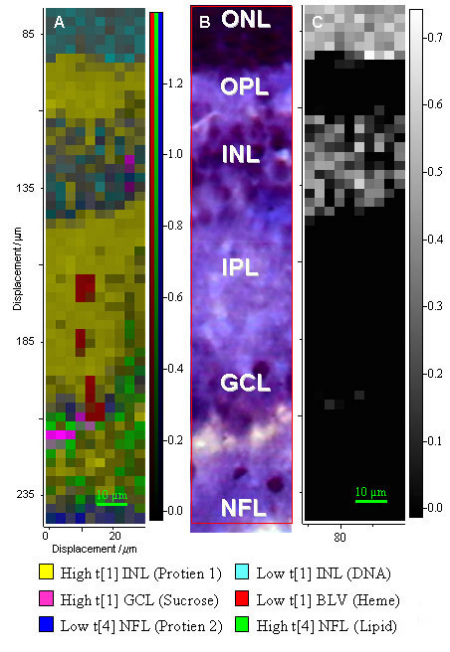
Comparison of Raman data with histological section. **A**: Raman map of the distribution of major spectral components in the inner segments of the area centralis of porcine retina. Principal component analysis was used to create reference spectra for linear combination of elements (LCE) analysis of the spectral data. Six major spectral contributors were selected for mapping; these were assigned to DNA, to two distinct proteins, fatty acid, heme, and sucrose (cyroprotectant medium). **B**: Optical image of the section following hematoxylin and eosin staining after Raman analysis of the same area. **C**: Monochrome Raman map of the DNA signal, allowing more sensitive detection of the DNA signals.

**Figure 7:**
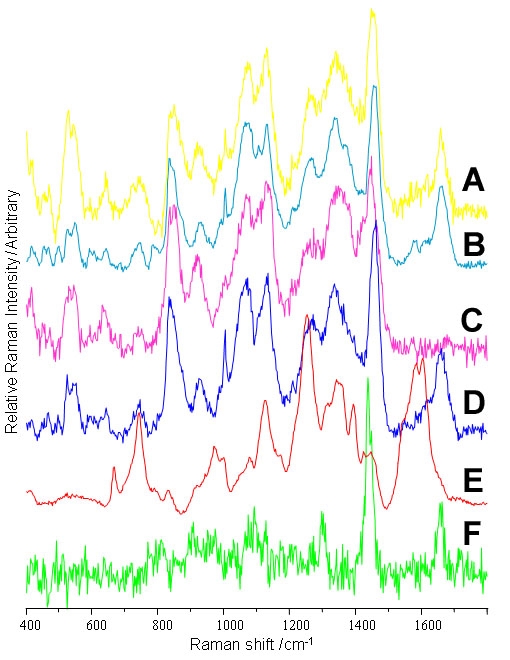
Major Raman signals (633 nm excitation) in the inner layers of porcine retina. Raman spectra, selected from each of the retinal layers were generated using the first significant principal component for that layer. Some components spanned a number of layers; consequently, the total number of spectral contributors mapped was six. **A**: High t [1] inner nuclear layer (INL; Protein 1), **B**: Low t [1] INL (DNA), **C**: High t [1] GCL (Sucrose), **D**: Low t [1] nerve fiber layer (NFL; Protein 2), **E**: Low t [1] blood vessel (BLV, Heme), **F**: High t [1] NFL (lipid). The Raman map in Figure 6 was generated from the linear combination of these reference spectra that accounted for each Raman spectrum. The color coding of the spectral signals is consistent with Figure 6.

PCA was employed to study the two principal sources of variation within each layer, i.e. the two predominant biochemicals in the respective layers. The distribution of the various biochemical constituents identified using PCA from the individual layers revealed that there was a considerable degree of homogeneity with many matching signals evident in more than one layer. This contrasts with the Raman analysis of photoreceptor outer segments, which displayed greater regional heterogeneity [10]. Principal components that were not significantly different between IPL, ONL, OPL, GCL, and NFL were combined and consequently the number of unique major principal components within all these layers was six. For simplicity the following paragraph in its entirety refers to the Raman map in Figure 6, with the spectral signals relating to each color code identified for Figure 7.

Clear banding was observed in the Raman map, which closely matched the stratified architecture of the retinal cross-section. The yellow-coded spectrum (Figure 7A), typical of protein and detected in the OPL, IPL, GCL, and NFL, dominated the inner retinal layers. The cyan-coded DNA signal (Figure 7B) was readily apparent in the INL [1] and exhibited a lower intensity than in the ONL, thus reflecting the lower nuclear density. Histological analysis revealed the characteristic pattern of scattered nuclei in the GCL, and mapping only the Raman-PCA scores relating to the DNA revealed scattered nuclei in the GCL; this was achieved by excluding the predominant signals, which overrode the weaker DNA signal. The red-coded heme signal (Figure 7E) was detected in isolated spots, forming a vertical line within the IPL, which is characteristic of vascular networks. Two distinct signals were observed in the innermost layers, with the green coded signal (Figure 7F) corresponding to fatty acid increasing within the GCL. The blue-coded signal (Figure 7D), corresponding to a protein distinct from the yellow-coded one, was observed in the nerve fiber layer.

### Sucrose

A proportion of the spectrum detected arose from the cryoprotectant medium, sucrose (Figure 7C). There was a significant increase in its level proceeding from the OPL to the NFL, though this was masked by the dominant cellular signals in Figure 7.

## Discussion

The proteins of the inner retina, specifically within the OPL, INL, IPL, GCL, and NFL exhibited less variation than those detected in the ONL, PIS, and POS [10], while the layers in the inner retina exhibited elevated contributions from lipids as compared to the ONL. These observations were consistent with the architectural composition of the inner retinal layers. It is composed of a characteristic network of neurons, their axons extend into and generate the OPL and IPL, thus leading to a relatively homogenous spectrum. Muller glia, which possess elaborate cytoplasmic processes that extend among the cells and plexiform layers, are likely to also contribute to the uniform Raman profiles, together with the extracellular matrix.

### Plexiform layers

The OPL and IPL, as expected, exhibited common Raman spectra (Figure 1 and Figure 6) since each layer performed a similar function; the formation of synapses to allow the transfer of the electrical and chemical signals from the photoreceptors. Since these regions were densely packed with synapses, they were likely to possess higher concentrations of a range of synaptic ion channel proteins, along with a complement of neurotransmitter receptor protein complexes and their analogous neurotransmitters. These distinct biomolecules would account for the different protein signal observed in these layers relative to the nuclear layers. However, the one minor difference between the two plexiform layers (Figure 1) does appear to reflect the differing proportion of lipids that occured within the OPL and IPL, with the IPL showing a higher contribution from fatty acid based lipids.

### Nuclear layers

The observed decrease in the DNA concentration detected in the INL (Figure 2 and Figure 6) mirrors the decrease in nuclear density relative to the high nuclear density of the ONL. Furthermore, since the pig ONL is composed of the nuclei from primarily three photoreceptor cell types, rod and medium and short wave cone cells [12] this generated a degree of cellular homogeneity. In contrast, the INL possesses nuclei from neuronal horizontal, bipolar, amarcrine and Muller glia, which exhibit a species-dependent degree of phenotypic heterogeneity [13]. Collectively, this degree of cellular complexity offers a plausible explanation for the observed differences in the intensities of the Raman signals relating to fatty acid based lipids and in the protein population (Figure 2). The fatty acid lipid population in the INL appeared to be relatively saturated and adopted a more crystalline structure under the conditions in which the samples were analyzed. This is discernible in the low ratio of the features at 1660 and 1440 cm^-1^ and the triplet of resolved bands in the 1400-1500 cm^-1^ region, respectively [14]. The presence of the band at 1405 cm^-1^ would suggest that the crystalline form of the lipid incorporated two chains per unit cell [15]. This was supported by the presence of the twist lipid band at 1299 cm^-1^ in the INL. In a more disordered lipid this feature would be expected to shift to around 1305 cm^-1^ [14].

### Melatonin metabolites

Metabolites of melatonin would be expected to exhibit intensity at common Raman shifts as they share a common molecular skeleton. Indeed the unknown spectrum (Figure 3) from the comparison of the IPL and GCL and of the GCL and NFL also had bands overlapping, or close to, these shared bands. Only three bands, which could be assigned to an indole ring (not present in the kynurenine) vibration, did not appear to carry over to the unidentified components. Relative Raman intensities are sensitive to a wide range of factors including physical state and environmental interactions in addition to the obvious factor of chemical changes.

Melatonin is synthesized from tryptophan within the GCL in the dark and is degraded in the light by receptors in the IPL to its kynurenine. Potentially, the kynurenine observed in the GCL and IPL could be attributed to the method of sample preparation employed in this study, where the sections were cut under standard laboratory lighting and exposed to air to ensure adherence of the sections to the slides prior to analysis. Analysis of the various stages of melatonin metabolism would require the preparation of tissue sections under red light in a controlled atmosphere and use with red laser such as the 633 and 785 nm lasers employed in this study.

### Blood Vessels

Numerous studies have established that Raman spectroscopy (particularly resonance enhanced methods) can be used to investigate the oxidation state and conformation of heme compounds [5]. The broad range of wavelength sensitive peaks observed in blood vessels (Figure 4) was not as well resolved as previously observed for cytochrome C (the part of the molecule enhanced by resonance is very similar to heme) in the PIS [10]. This was not unexpected since hemoglobin is much more complex and is subjected to various oxidative states of the hemes that generate a more complex mixture of oxidation states and conformations. This is likely to be a factor since the methods employed were not designed for the specific study of oxidation or conformation of the hemes. Indeed the difference between the blood vessels and the clotted blood observed in Figure 4 may be traced to differences in redox conditions during the respective preparation methods. The magnitude of enhancement (Figure 4) is also influenced by the oxidation state and conformation of the heme as this affects the wavelengths of light that the molecule absorbs, the central factor governing resonance enhancement. The pattern of bands which are enhanced changes between 633 and 514 nm. This arises as different chemical bonds absorb these two wavelengths, and only the Raman signal from the chemical bond involved in the specific absorption is enhanced. As has been previously demonstrated [5], control of specimen preparation and handling would allow investigation of the oxidation states and conformations of the hemes and cytochromes within the retina, while careful choice of excitation wavelength allows selective study of specific bonds within the heme group.

### Nerve fiber layer

The part of the signal that changes between the GCL and NFL matches that discussed above (melatonin metabolites section), in relation to kynurenine. The reduction of the "kynurenine" signal in the NFL (Figure 5) is not surprising as this layer consists primarily of unmyelinated axons that convey nerve impulses to the optic nerve head, and are not involved in the metabolism of melatonin. The remaining protein signal (Figure 6 and Figure 7F) observed could arise from the extracellular matrix, which is a complex mixture of proteins and proteoglycans.

### Principal components analysis

The appearance of similar signals in the distinct stratified layers of the retina (Figure 6) was not surprising as various cell types span several layers. For example, amacrine cells form complex patterns of synaptic connections among ganglion and bipolar cell populations. The yellow coded signal may be tentatively assigned to the fastigiate Muller cell population since these cells have extensive cytoplasmic processes and give rise to the inner and outer limiting membranes. The nerve fiber layer consists of unmyelinated nerve fibers, which have a distinct protein population of extracellular matrix proteoglycans, which was clearly indentifiable from the Raman signal; this signal was localized (dark blue shades, Figure 6) to the nerve fiber layer.

In general, the mammalian retinal vasculature adopts a characteristic three-dimensional distribution; the major vessels radiate along the outer NFL. Minor vessels ramify tangentially and perpendicularly through the NFL and GCL to generate vascular networks in the IPL, and INL that terminate in a capillary network at the OPL interface with the photoreceptor peduncles. Raman analysis has identified portions of a vertical capillary, which are encoded red on the map (Figure 6).

The distribution of the cyan DNA signal (Figure 6) matched the distribution of the nuclei within the retinal section. A band of DNA signals was localized in the INL, which showed a marked decrease in intensity with respect to the ONL, which agrees well with the known differences in the densities of nuclei between the two layers. As expected, isolated DNA signals were observed in the GCL, and this was consistent with the scattered relatively sparse ganglion cell population. The increase in the green fatty acid signal at the bottom of the GCL and into the NFL reflected the increasing unmyelinated neuron density in this region.

The increase in the sucrose signal detected across the inner layers most probably reflected the fact that sucrose, present as the mounting medium, has infiltrated the large cytoplasmic volume. This is derived from the extensive neuronal processes comprising the array of neuronal and non-neuronal cell types that dominate the inner layers [13].

### Non Raman background

It is clear from the figures of the raw spectral data that, in addition to the changes in the sharper Raman bands, the broad featureless background varies significantly. The Raman effect upon which the spectroscopic method is based is highly inefficient (ca. 10^-6^ of incident photons are affected). Consequently, any other visible light phenomena, such as fluorescence and elastic scattering, can give significant intensity to the recorded data. In this data there are clearly minor changes in this featureless background, but due to its low information content it is usually removed prior to quantitative analysis [1]. In this paper the manual comparisons have been performed on unbaselined data in order to avoid the problem of spurious artifacts that can occur when subtracting data that has had its non-Raman baseline removed.

### Conclusion

This study has further demonstrated the potential of Raman microscopy to investigate the distribution of various biochemicals within porcine retina, more specifically, protein populations, lipids, hemes, and DNA within the inner layers. Raman signals for the molecular species encountered in the inner layers were distinct from the outer layers [10], as expected from the distinctly different population of cells in the inner and outer layers. While all three of the probe wavelengths employed during the present investigation have proven suitable for investigating lipids, proteins, and DNA, the spectral data indicated that 633 nm is an excellent wavelength for investigating the hemes of the capillaries. This wavelength appears to give the largest scattering enhancement of the three wavelengths employed. Finally, the construction of a Raman map provides an informative overview of the complexity of the biomolecules in the inner retina and effectively demonstrates the potential of Raman spectroscopy to study retinal structure and its biochemical composition, while at the same time displaying considerable promise in the future for mapping the progression of retinal pathologies.
